# 

**Published:** 1910-01

**Authors:** 


THE ROCKEFELLER COMMISSION ON HOOK WORM will meet in Atlanta, January 16-18, inst. Brumby and Cummings of the Texas Board of Health will go as delegates.

---

YE EDITOR of ye "Red Back" wishes all its subscribers a happy New Year. He is sure they all wish him a prosperous New Year, and he takes the liberty of suggesting that attention to his little subscription bills lately sent out and a prompt remittance will contribute to his health, happiness and prosperity with a capital P. Selah.

